# Renal Failure Following Traumatic Bladder Rupture: A Case Report

**DOI:** 10.7759/cureus.117955

**Published:** 2026-10-15

**Authors:** Douaa Kaouani, Mariam Lagrine, Rabiy Elqadiry, Houda Nassih, Aicha Abourrahouat, Imane Aitsab, Sara Ouassil, Hussein Choukri Ahmanna, Hicham Jalal, Salma Foura

**Affiliations:** 1 Department of Pediatrics, University Hospital Center Mohammed VI, Marrakech, MAR; 2 Department of Radiology, University Hospital Center Mohammed VI, Marrakech, MAR; 3 Department of Pediatric Surgery, University Hospital Center Mohammed VI, Marrakech, MAR

**Keywords:** acute renal failure, ascites, bladder rupture, ct scan, trauma

## Abstract

When the bladder ruptures into the peritoneal cavity, urine rich in creatinine and urea can accumulate freely within the abdomen. The resulting rise in serum creatinine and urea therefore stems from passive reabsorption of these solutes across the peritoneal membrane rather than from an actual decline in kidney function, and the calculated glomerular filtration rate (GFR) returns to normal shortly after the bladder is repaired and the peritoneal cavity washed out.

We describe a previously healthy six-year-old girl who developed pseudo-renal failure from an intraperitoneal bladder rupture sustained one day earlier during a fall from standing height. She presented to the emergency department with severe generalized abdominal pain and macroscopic hematuria with preserved diuresis. The examination revealed a patient stable hemodynamically and respiratorily, with ascites and general tenderness without abdominal guarding, the pain being most pronounced in the suprapubic area. Blood tests showed elevated creatinine levels at 61.64 mg/L and a urea level at 2.66 g/L. The abdominal and pelvic CT scan revealed a large amount of ascites, with identification of the distal end of the urinary catheter in the intra-abdominal cavity through a defect in the upper wall of the bladder measuring 9 mm in diameter, and extravasation of contrast medium during the urinary phase. However, the liver, spleen, pancreas, kidneys, and ureters appeared normal. Through this observation, we raise awareness of the possibility of bladder rupture in the presence of acute renal failure even without a history of severe trauma.

## Introduction

When a ruptured bladder leaks urine into the peritoneum, the resulting ascites can raise serum urea and creatinine through simple diffusion across the peritoneal lining, producing a biochemical picture that is easily mistaken for genuine acute kidney injury; because this mechanism is not widely appreciated, the diagnosis is frequently overlooked [1]. In the pediatric population, the intraperitoneal pattern is clearly more common since their bladder sits higher and more intra-abdominally than in adults, and cases have been documented even after apparently trivial trauma [2,3]. Here, we present a six-year-old girl who developed this pseudo-renal picture after a minor fall, illustrating why clinicians should suspect an occult bladder injury whenever biochemical evidence of renal impairment seems out of proportion to the reported mechanism of injury.

## Case presentation

A previously healthy six-year-old girl presented to the emergency department with severe generalized abdominal pain. A urinary catheter was placed when the patient was admitted to measure urine output, which showed macroscopic hematuria and preserved diuresis at 2 cc/kg/day. Her recent history included a fall from standing height one day prior; this procedure does not have a direct impact on the lower abdomen or the suprapubic area. The physical examination revealed a conscious child, stable hemodynamically and respiratorily, afebrile, with a normal heart rate of 94 beats per minute and a blood pressure of 100/60 mmHg. Urinalysis showed 2+ protein and 3+ hematuria. The abdomen was distended with ascites and generalized tenderness without abdominal guarding, the pain being most pronounced in the suprapubic area. There was neither rash nor bruising. The extremities were warm and well-perfused, without edema.

On assessment, hemoglobin was 14.6 g/dL; total white blood cell count was 22,550 cells/µL; differential count showed neutrophils 90%, lymphocytes 5.4%, eosinophils 0%, and monocytes 4.4%; platelet count was 452,000/µL. Urinalysis showed 1,153,000 leukocytes/mL and 27,783,000 red blood cells/mL in the same sample. Arterial blood gas analysis showed pH 7.31, bicarbonate 8 mmol/L, and pCO₂ 22.2 mmHg. Urea was 2.66 g/L, creatinine was 6.164 mg/dL, sodium was 137 mmol/L, potassium was 3.4 mmol/L, and calcium was 92 mg/L (Table 1). A median laparotomy was performed due to the unavailability of laparoscopic facilities at our institution. During surgical exploration, a 3 cm laceration at the dome of the bladder was repaired, and the urinary ascites was evacuated. The child's postoperative course proceeded without incident, with normalization of serum creatinine and urea.

**Table 1:** Laboratory findings on admission with reference ranges for a 6-year-old child.

Parameter	Patient value	Reference range
Hemoglobin	14.6 g/dL	11.5-15.5 g/dL
White blood cell count	22,550/µL	5,000-14,500/µL
Neutrophils	90%	40%-60%
Lymphocytes	5.4%	25%-45%
Eosinophils	0%	0%-5%
Monocytes	4.4%	2%-8%
Platelets	452,000/µL	150,000-450,000/µL
Urine leukocytes	1,153,000/mL	<10,000/mL
Urine red blood cells	27,783,000/mL	<10,000/mL
Arterial pH	7.31	7.35-7.45
Bicarbonate (HCO₃⁻)	8 mmol/L	22-26 mmol/L
pCO₂	22.2 mmHg	35-45 mmHg
Urea	2.66 g/L	0.15-0.45 g/L
Creatinine	6.164 mg/dL	3-7 mg/L
Sodium	137 mmol/L	135-145 mmol/L
Potassium	3.4 mmol/L	3.5-5.0 mmol/L
Calcium	92 mg/L	90-110 mg/L

Due to the history of trauma and concern for occult injury to the solid abdominal viscera, an abdominal CT scan with intravenous contrast, including delayed and excretory-phase imaging, was performed, revealing a large volume of ascites with identification of the distal tip of the urinary catheter intra-abdominally through a parietal defect in the upper wall of the bladder measuring 9 mm in diameter, with contrast extravasation during the urinary phase; the liver, spleen, pancreas, kidneys, and ureters appeared normal (Figures 1, 2).

**Figure 1:**
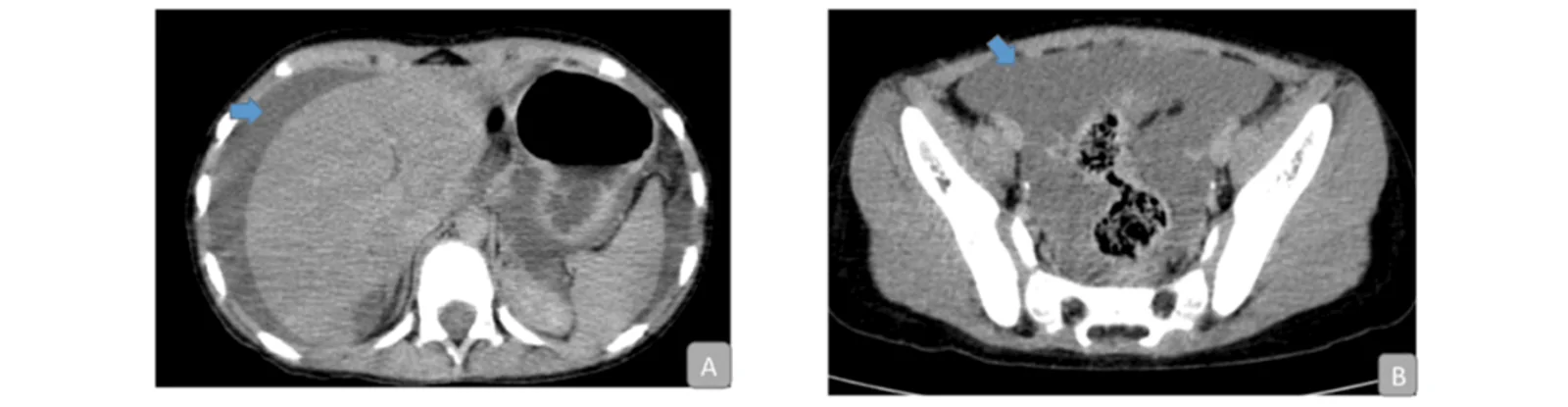
Abdominopelvic CT: axial slice at the abdominal level (A) and pelvic level (B) after contrast injection showing a very significant peritoneal effusion.

**Figure 2:**
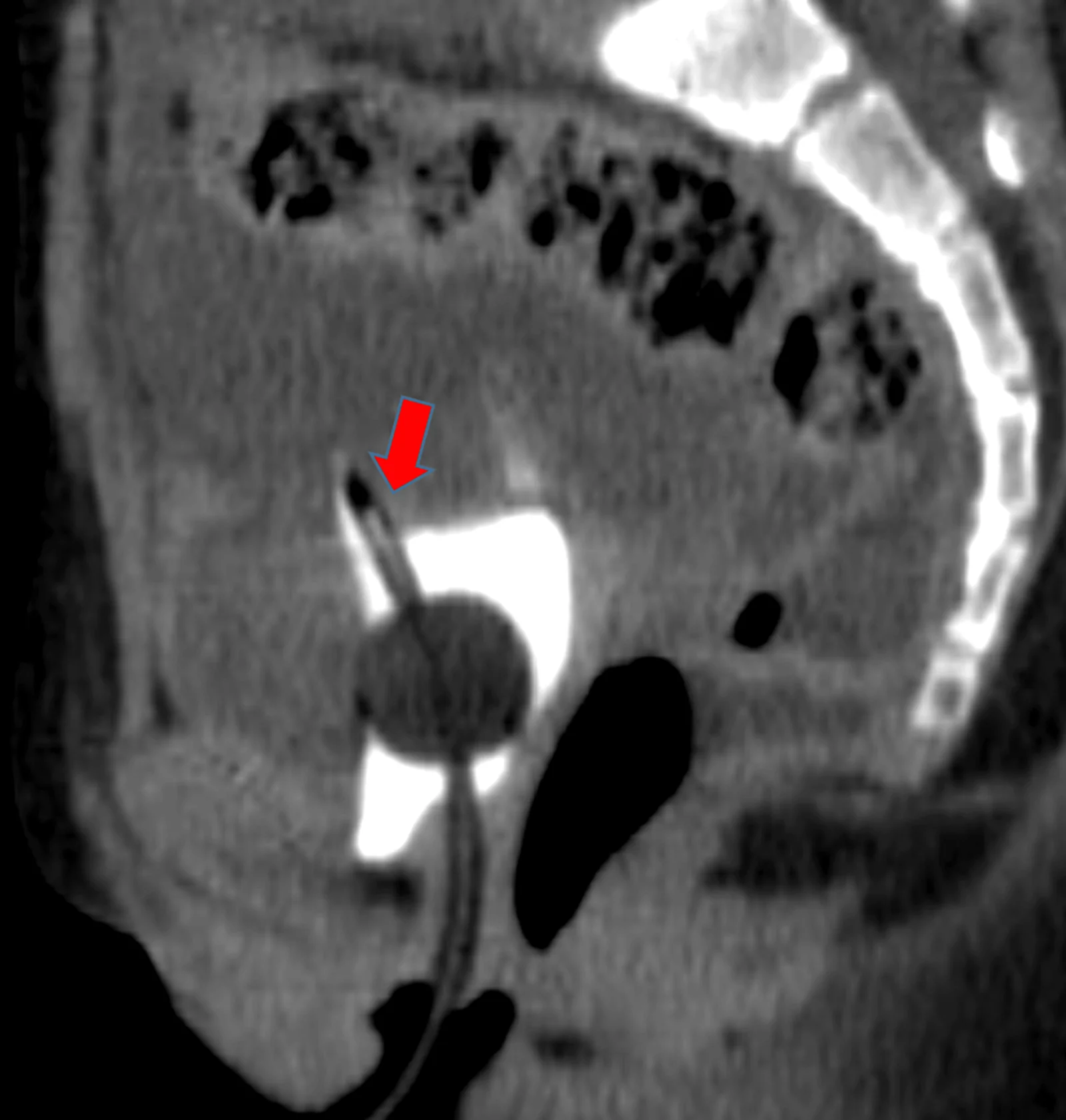
Abdominopelvic CT scan (sagittal view): the distal tip of the urinary catheter is seen inside the abdomen through a 9 mm defect in the upper wall of the bladder, with contrast medium leaking during the urinary phase.

A median laparotomy was performed due to the unavailability of laparoscopic facilities at our institution. During surgical exploration, a 3 cm laceration at the dome of the bladder was repaired, and the urinary ascites was evacuated. The child's postoperative course proceeded without incident, with normalization of serum creatinine and urea.

## Discussion

Bladder rupture without a traumatic cause is rare, affecting roughly one person in 126,000, and trauma accounts for the great majority of cases overall (about 96%) [1]. The site of rupture tends to differ by mechanism: spontaneous and iatrogenic injuries are usually intraperitoneal, whereas traumatic ruptures, especially those linked to pelvic fractures, are more often extraperitoneal. Because the bladder dome is the thinnest and least supported part of the organ, it gives way first when a full bladder is subjected to a sudden rise in intravesical pressure [2].

The predominance of intra- or extraperitoneal rupture in adults remains an unresolved issue. Still, in the pediatric population, the intraperitoneal pattern is clearly more common, reflecting the fact that a child's bladder lies largely within the abdominal cavity rather than tucked behind the pelvic bones, as in adults [3,4]. Occasionally, both compartments are breached at once [3].

No single clinical sign reliably identifies a bladder injury. Hematuria, painful voiding, and urinary retention are frequent findings but are not specific to rupture, and even patients with confirmed intraperitoneal injury do not consistently show abdominal pain or hemodynamic compromise. When the defect is large enough, the bladder may fail to fill at all, so patients can lose both the urge and the ability to void [4,5].

When the bladder ruptures intraperitoneally, urine collects freely in the abdominal cavity as urinary ascites. Because this fluid contains far more creatinine and urea than plasma does, a steep concentration gradient forms across the peritoneal membrane, and nitrogenous waste diffuses back into the bloodstream, a process sometimes termed reverse peritoneal dialysis. This condition is what produces the electrolyte disturbances (notably hyponatremia and hyperkalemia) and the spuriously elevated creatinine with an apparently reduced estimated glomerular filtration rate (GFR) that we observed in our patient, even though her kidneys themselves were undamaged [6].

A picture of renal failure caused by bladder rupture, as seen in our patient, is uncommon overall [7-9]. Besides blunt abdominal trauma, published cases attribute pseudo-renal failure to a variety of other insults to the bladder wall: perforation during laparoscopic surgery [6], rupture of a bladder diverticulum [10], infection [11,12], tumor invasion [13], and, less commonly, spontaneous perforation following pelvic radiotherapy [14].

A useful biochemical clue is the ascitic-to-serum creatinine ratio: a value above one points toward an intraperitoneal urinary leak and can, in principle, be obtained by paracentesis. In practice, however, performing a diagnostic puncture is not always feasible or justified in the emergency setting, and we were unable to obtain ascitic fluid for analysis in our patient.

Contrast-enhanced abdominal CT remains the imaging modality of choice for assessing intra-abdominal trauma, and radiologists interpreting these scans should keep intraperitoneal bladder rupture on their list of possible explanations whenever ascites is accompanied by renal impairment. Two findings should raise suspicion: a bladder that fails to distend as expected, and contrast material seen outside the bladder lumen within the pelvis [6].

For a hemodynamically stable child without major associated organ injury, laparoscopy is a reasonable alternative to open laparotomy, provided the surgical team can convert quickly if the situation demands it. Its benefits include thorough visualization of the bladder and adjacent structures, a secure repair of the defect, effective irrigation of the peritoneal cavity, and earlier resumption of oral intake [15].

## Conclusions

When patients present with acute abdominal pain, hematuria, and biochemical signs of renal failure such as increased serum urea and creatinine, the physician should consider the possibility of an intraperitoneal bladder rupture. In our patient, surgical repair via laparotomy was required to close the bladder defect and evacuate the urinary ascites. Renal function normalized following bladder repair and peritoneal lavage.

This case underlines the value of maintaining a high level of suspicion for bladder injury even when the reported trauma appears minor, since clinical presentation alone can be misleading. Early identification through appropriate imaging can prevent an unnecessary diagnostic workup for primary renal disease and allow timely surgical intervention. As illustrated here, prompt recognition and repair are generally followed by a favorable outcome, with rapid resolution of the biochemical abnormalities.
